# Wound healing and signaling pathways

**DOI:** 10.1515/biol-2025-1166

**Published:** 2025-09-01

**Authors:** Zhe Liu, Yudong Fang

**Affiliations:** Shanghai TCM-Integrated Hospital, Department of Vascular Diseases, Shanghai University of TCM, No. 230, Baoding Road, Hongkou District, Shanghai, Shanghai Municipality, 200082, China

**Keywords:** wound healing, signaling pathway, crosstalk, therapeutic strategies

## Abstract

Wound healing is a precisely regulated dynamic process in which signaling pathways play a central role. This article provides a comprehensive review of the signaling pathways involved in wound healing, emphasizing their roles in inflammation, vascular regeneration, cell proliferation, and extracellular matrix remodeling. We further discuss the crosstalk between these pathways and their contributions to wound healing dysregulation. Finally, we explore emerging therapeutic strategies targeting these pathways, including small-molecule inhibitors, gene therapy, and biologics, summarizing their preclinical and clinical efficacy. By elucidating the molecular mechanisms underlying wound healing and potential interventions, this review aims to provide valuable insights for future research and translational applications in wound healing.

## 1 Introduction

Wound healing is a dynamic and multifaceted biological process that requires the precise coordination of inflammation, angiogenesis, cell proliferation, and extracellular matrix remodeling. While acute wounds progress through a well-defined sequence of hemostasis, inflammation, proliferation, and remodeling, chronic refractory wounds remain stalled in one or more of these phases, leading to prolonged tissue damage and increased susceptibility to infection. The condition of wound healing is usually also associated with diabetes, ischemia, venous insufficiency, and pressure ulcers. These factors can lead to difficulties in wound healing, significantly reduce the quality of life of patients, and impose a heavy burden on healthcare [1].

The persistent failure of wound healing is largely driven by dysregulated signaling networks that govern cellular responses to injury. Key pathways such as mitogen-activated protein kinase (MAPK), phosphatidylinositide 3-kinases (PI3K)/AKT, TGF-β, NF-κB, Wnt/β-catenin, Hippo-Yes-associated protein (YAP), and Notch are involved in regulating immune responses, vascular remodeling, fibroblast activation, and extracellular matrix turnover. Disruptions in these pathways contribute to excessive inflammation, inadequate angiogenesis, fibroblast dysfunction, and pathological fibrosis, ultimately leading to wound chronicity.

In recent years, increasing efforts have been directed toward identifying molecular targets within these pathways to develop novel therapeutic strategies. Emerging approaches, including small-molecule inhibitors, gene therapy, and biologics, hold promise for modulating key signaling pathways and restoring normal wound healing processes. However, challenges remain in translating these therapies into clinical practice due to issues related to delivery efficiency, off-target effects, and long-term safety.

This review aims to provide a comprehensive analysis of the key signaling pathways in wound healing, their pathological implications, and their interconnections. Furthermore, we discuss current and emerging therapeutic strategies targeting these pathways, summarizing their preclinical and clinical evidence. By elucidating these mechanisms, we hope to contribute to the development of more effective treatments for wound healing.

## 2 Overview of mechanisms involved in wound healing

### 2.1 Inflammatory response

Inflammation is an essential initial phase of wound healing, playing a pivotal role in eliminating pathogens and orchestrating tissue repair. Upon injury, damaged tissues release damage-associated molecular patterns and pro-inflammatory cytokines such as tumor necrosis factor-alpha (TNF-α), interleukin (IL)-1 beta, and IL-6, which recruit immune cells to the wound site. Neutrophils are the first responders, producing reactive oxygen species and antimicrobial peptides to clear invading pathogens. This is followed by monocyte infiltration and their differentiation into macrophages, which transition from an inflammatory (M1) to a reparative (M2) phenotype, facilitating tissue regeneration. However, in wound healing, persistent activation of the inflammatory response leads to excessive cytokine secretion, sustained neutrophil infiltration, and macrophage dysfunction, ultimately impairing wound healing and promoting tissue damage.

### 2.2 Angiogenesis

Angiogenesis, the formation of new blood vessels from pre-existing vasculature, is critical for delivering oxygen and nutrients to regenerating tissues. This process is tightly regulated by pro-angiogenic factors such as vascular endothelial growth factor (VEGF), fibroblast growth factors, and angiopoietins, while anti-angiogenic factors such as thrombospondins and endostatin counterbalance their effects. Under normal conditions, angiogenesis is activated in response to hypoxia and tissue injury, promoting capillary sprouting and endothelial cell (EC) migration. However, in wounds healing, dysregulated angiogenic signaling – often due to prolonged inflammation, endothelial dysfunction, or metabolic disturbances – leads to either insufficient or aberrant vascularization, resulting in tissue hypoxia and impaired healing.

### 2.3 Cell proliferation and migration

The proliferation and migration of keratinocytes are crucial for wound healing and repair. Epidermal stem cells, located in the basal layer of skin, are specific stem cells of skin tissue with infinite proliferation and differentiation potential. As the differentiation source of epidermal cells of all layers in the process of wound epithelialization, the proliferation and differentiation of epidermal stem cells contribute to the renewal of epidermal cells [2]. The interaction between cells and the extracellular matrix plays a crucial role in influencing stem cell differentiation through various cytokines that convey information.

### 2.4 Extracellular matrix remodeling

Extracellular matrix is a complex network composed of multiple macromolecules around cells, which can provide a suitable microenvironment for cells, mainly containing collagen, fibrin, elastin, laminin and other substances. As the main component of the extracellular matrix, collagen is distributed in various organs and tissues. Extracellular matrix remodeling is particularly important in the later stage of wound healing, and collagen synthesis and degradation are important factors affecting wound healing. Studies have shown that improving the content and stability of collagen can accelerate wound healing [3].

## 3 Overview of signaling pathways

### 3.1 MAPK signaling pathway

**Figure 1:**
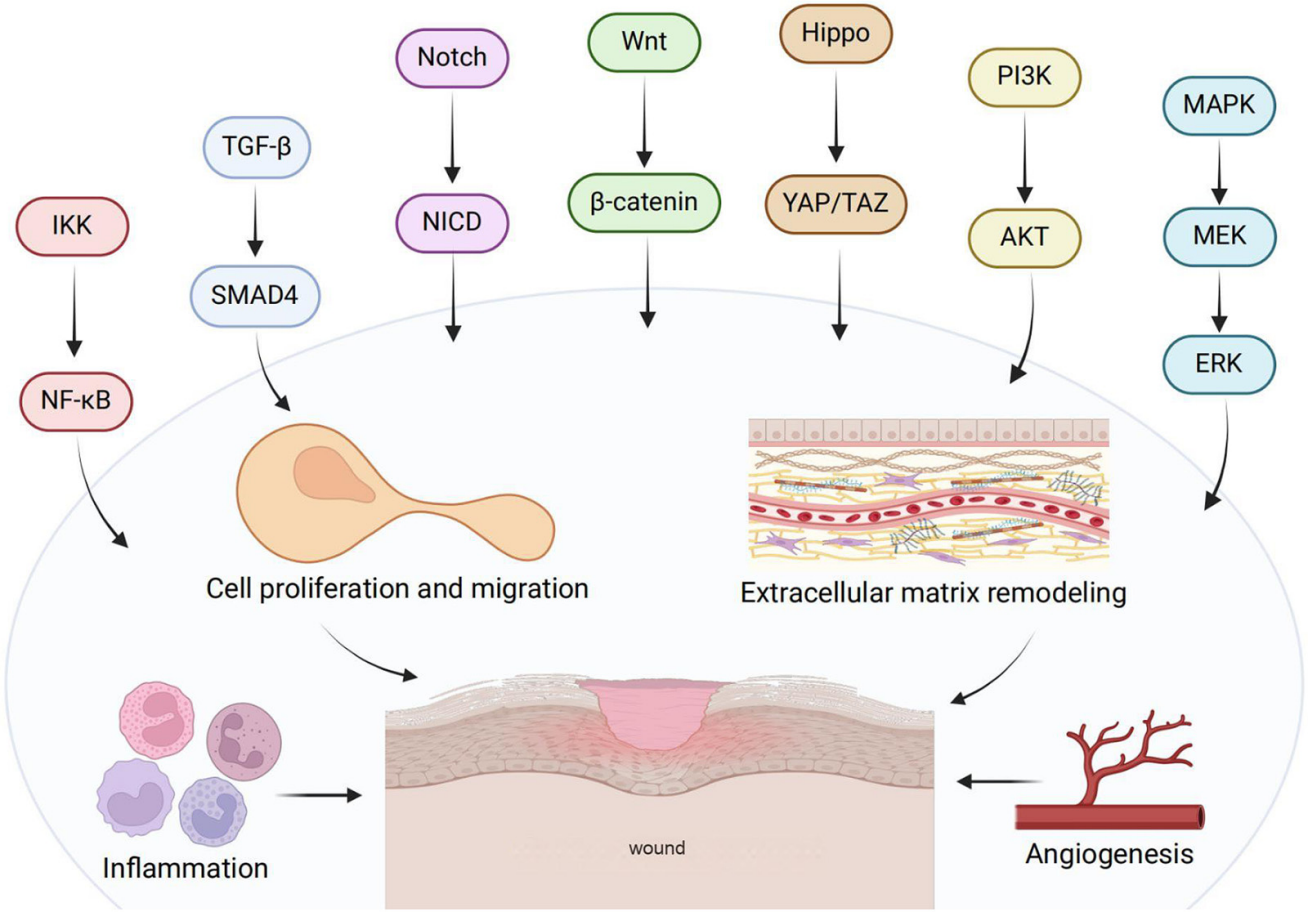
Schematic diagram showing the biological and pathological processes and signaling pathways underlying the wound healing.

The MAPK pathway involves three-stage signaling processes: MAPK, MAPK kinase (MEK or MKK), and the kinase of MAPK kinase (MEKK or MKKK) (Figure 1). These three kinases can be activated sequentially and jointly regulate colossal amounts of important physiological/pathological effects such as cell growth and differentiation and stress and inflammatory responses. The MAPK pathway covers four main branching routes: ERK, JNK, P38/MAPK, and ERK5. The MAPK signaling pathway can induce cell migration and proliferation, so as to accelerate wound healing.

#### 3.1.1 The MAPK signaling pathway can affect wound healing by affecting cell proliferation and repair

p38 is the most important member of the MAPK family in regulating inflammatory response, and the p38 signaling pathway is closely related to inflammatory response and apoptosis. Studies have shown that the p38/MAPK2 signaling pathway can regulate the expression of inflammatory factors and participate in wound healing, therefore plays a key role in the process of wound repair [4,5,6,7]. JNK is a stress-activated protein kinase in the cytoplasm. As a key molecule in the signal transduction process of various stressors, JNK plays an important role in osmotic pressure changes and inflammatory responses of cells [8]. The JNK signaling pathway is an important member of the MAPK inflammatory pathway. Studies have shown that JNK inhibitors can reduce the expression of related inflammatory factors in infected tissues, thereby alleviating the inflammatory response [9]. ERK signaling pathway is a classic MAPK signal transduction pathway, which not only participates in the regulation of cell proliferation and differentiation but also participates in the regulation of cell morphology and the redistribution of skeleton through phosphorylation of cytoskeletal components in the cytoplasm.

#### 3.1.2 The MAPK pathways can affect wound healing by affecting vascular regeneration

Hou et al. [10] found that the microvascular density of wound was significantly increased in rats with open skin wounds treated with vaccinin, and the lymph node proteins and receptors related to the angiogenesis signaling pathway were activated (increased expression of phosphorylated ERK), confirming that the process was mediated by the MAPK/ERK signaling pathway. Based on the analysis of human wound cell culture medium, Geng et al. [11] found that activating the MAPK/ERK signaling pathway could promote the increase of VEGF level, suggesting that the activation of the MAPK/ERK signaling pathway has a positive effect on wound healing.

#### 3.1.3 The MAPK pathways can affect wound healing by affecting extracellular matrix remodeling

Wang et al. [12] found that activating the ERK/MAPK signaling pathway can increase the expression of matrix metalloproteinase in dermal fibroblasts and regulate extracellular matrix remodeling, thereby promoting wound repair. Ji et al. [13] demonstrated that RUNX1 transcriptionally activates osteopontin (OPN), which suppresses keratinocyte migration and ECM degradation by inhibiting MAPK pathway activity. Silencing OPN or downregulating RUNX1 releases this inhibition, thereby activating ERK/p38 MAPK signaling to enhance HaCaT cell proliferation, migration, and MMP-mediated ECM remodeling, ultimately accelerating burn wound healing.

#### 3.1.4 The MAPK signaling pathway can affect wound healing by affecting cell proliferation and repair

Konstantinou et al. [14] studied the mechanism of microcurrent in treating chronic wounds and promoting their healing. The *in vitro* experiment suggested that microcurrent stimulated the phosphorylation of ERK1/2 and p38 kinases related to the MAPK signaling pathway in fibroblasts, induced cell migration and proliferation, and thus accelerated wound healing. Zhang et al. [15] confirmed through *in vivo* and *in vitro* experiments that the P38/MAPK signaling pathway in damaged epidermal keratinocytes induced microtubule (MT)-associated protein 4 (MAP4) phosphorylation under hypoxia, which has the ability to regulate cell migration and proliferation, and at the same time, it induced MT decomposition and accelerated keratinocyte proliferation and migration, which promoted wound healing.

### 3.2 PI3K/AKT signaling pathway

PI3K is a kind of lipid kinase in the cytoplasm, which is involved in the signal transduction, transport, and metabolism of the cell membrane. PI3K is a dimer composed of regulatory subunit P85 and catalytic subunit P110. When it binds to growth factor receptors (such as EGFR), it can change the protein structure of AKT and activate it and, at the same time, activate or inhibit a series of downstream substrates such as apoptosis-related proteins Bad and Caspase9 through phosphorylation, thus regulating cell proliferation, differentiation, apoptosis, and migration. What is more, protein kinase B (AKT) can also activate IKK and produce crosstalk with the NF-κB pathway. The downstream target of PI3K/AKT is mammalian target of rapamycin (mTOR), and the downstream transcription factors of mTOR include HIF1α, c-MYC, FoxO, and other star molecules. The PI3K/AKT signaling pathway is closely related to wound healing [16].

#### 3.2.1 The PI3K/AKT pathways can affect wound healing by affecting vascular regeneration

PI3K can induce the phosphorylation of AKT by catalyzing the phosphorylation of phosphatidylinositol diphosphate, thereby regulating the transcriptional level of downstream endothelial nitric oxide synthase (eNOS) and stimulating the synthesis and release of nitric oxide [17]. As a potent angiogenic medium, nitric oxide plays a role in regulating angiogenic factors, not only stimulating neoangiogenesis but also participating in regulating the proliferation, invasion, apoptosis, and lumen formation of ECs, which can significantly delay wound healing [18]. Yuan et al. [19] adopted different concentrations of cinnamaldehyde to interface with human umbilical vein colorectal cells (HUVECs) and verified that cinnamaldehyde could induce the phosphorylation of AKT and eNOS, activating and up-regulating PI3K, so as to accelerate the angiogenesis and wound healing. Dong et al. [20] proved through an *in vitro* experiment that paeoniflorin could enhance the angiogenesis ability of HUVECs and facilitate wound healing by means of the PI3K/AKT signaling pathway.

#### 3.2.2 The PI3K/AKT signaling pathway can affect wound healing by influencing inflammatory responses

Yu et al. [21] studied the mechanism of insulin on macrophages phenotype conversion by establishing a burn wound in diabetic rats model and human monocyte THP-1 model and found that local injection of low-dose insulin around the wound could promote macrophages to change from proinflammatory (M1) phenotype to anti-inflammatory (M2) phenotype through the PI3K/AKT signaling pathway, as to accelerate wound healing.

#### 3.2.3 The PI3K/AKT pathways can affect wound healing by affecting extracellular matrix remodeling

Wang et al. [22] studied the effect of hypoxic adipose stem cells’ exosomes (HypadSCs-ExO) on wound healing by establishing a human fibroblast model and full-thickness skin resection in a diabetic nude rat model. The results suggested that relying on PI3K/AKT signaling pathway, HypADSCs-exo may regulate the proliferation and migration of fibroblasts and the secretion of inflammation, vascular-associated growth factors and extracellular matrix, and accelerate the high-quality wound healing in diabetic targets. Zhang et al. [23] studied the wounds of mice with whole-layer skin incision and found that activating the PI3K/AKT signaling pathway could promote the proliferation and migration of fibroblasts, and collagen deposition, thereby accelerating wound repair.

#### 3.2.4 The PI3K/AKT signaling pathway can affect wound healing by affecting cell proliferation and repair

Li et al. probed into the impact and mechanism of Human amniotic mesenchymal stem cells (hAMSCs) on burn wound healing through *in vivo* and *in vitro* experiments, which manifested that hAMSCs inhibited cell apoptosis by activating the PI3K/AKT signaling pathway, and this signaling pathway could activate glycogen synthase kinase-3 beta (GSK-3β)/β-catenin signaling pathway to promote cell proliferation and in turn achieve wound injury treatment [24,25].

### 3.3 TGF-β signaling pathway

TGF-β family is a kind of secretory polypeptide signaling molecule, which is involved in the regulation of cell proliferation, differentiation, apoptosis, and wound healing. Smad is usually the downstream effector transcription factor of the TGF-β signaling pathway, and TGF-β1/2/3 and BMP (bone morphogenetic protein) are the signal sources of this pathway. Studies have shown that inhibition of TGF-β signal transduction could accelerate the closure of scarless wound [26]. Among them, TGF-β1 is one of the main factors affecting wound healing and scar formation. TGF-β1 can stimulate fibroblast proliferation and promote the transformation of stromal cells into fibroblasts. TGF-β1 can also promote the transformation of fibroblasts into myofibroblasts, which is beneficial to wound contraction and early wound closure. Besides, TGF-β1 can stimulate the proliferation of vascular ECs and promote early vascular remodeling, which is conducive to granulation formation [27,28,29]. Smad protein is an intermediary molecule that conducts the extracellular TGF-β signal through the cytoplasm to the nucleus. The activation of the TGF-β1/Smad3 signaling pathway can stimulate the synthesis of other fibrous proteins, promote the proliferation and phenotypic transformation of fibroblasts, inhibit fibroblast apoptosis, and promote the synthesis of various extracellular matrices, which are of great significance for wound healing.

#### 3.3.1 TGF-β pathways can affect wound healing by affecting extracellular matrix remodeling

Shi et al. constructed an *in vitro* cell model and an *in vivo* animal model of ischemic ear wound to study the effects of clinical-grade platelets exosome products (PEP) developed and injectable surgical fibrin sealant (TISSEEL) on chronic ischemic wounds. The results showed that TISSEEL–PEP regulated the TGF-β pathway, including SMAD2, RAS, and other pathways, and promoted epithelialization enhancement, fibroblast activation, and collagen production, thus promoting ischemic wound healing. Tang et al. [30] also studied the effectiveness of Ginsenoside Rg3 in the treatment of scars through *in vitro* experiments, and the results showed that Rg3 could inhibit fibroblasts proliferation, angiogenesis, and collagen synthesis through TGF-β/Smad and ERK signaling pathways, so as to promote the perfect wound healing and effectively inhibit scar growth. Pan et al. [31] studied the relationship between angiogenin and scars by cultivating scar fibroblast model, and the results suggested that the expression of angiopoietin was negatively correlated with the severity of burn scars, that is, the increase of angiopoietin inhibited the expression level of TGF-β1/Smad2 and vice versa, which further confirmed that the excessive scar hyperplasia may be lightened and wound healing may be headlined via this mechanism [32,33].

#### 3.3.2 The TGF-β pathways can affect wound healing by affecting vascular regeneration

Miscianinov et al. [34] explored the relationship between the TGF-β pathway and ROtung-to-mesenchymal transition (EndMT) by establishing an EC model, and the results showed that high expression level of Mir-148b enhanced EC proliferation and migration and *in vitro* vascular formation by regulating TGFB2 and SMAD2, which are targeted genes in the TGF-β signaling pathway, and at the same time, it weakened the EndMT process and accelerated wound closure.

### 3.4 Wnt signaling pathway

Wnt signaling pathway is closely related to wound repair, involving the proliferation and migration of fibroblasts and keratinocytes, extracellular matrix and collagen contraction, angiogenesis, etc. [35,36,37,38,39]. Generally, the Wnt pathway mainly refers to the classical signaling pathway mediated by β-Catenin, which is an important biomarker for detecting Wnt activation. The content of β-catenin in the cytoplasm directly affects the proliferation and differentiation of epidermal stem cells. The higher the content of β-catenin, the stronger the differentiation and proliferation abilities of epidermal cells. β-Catenin can promote the development of skin and its appendages, wound angiogenesis, and epithelial remodeling, thereby accelerating wound healing [40]. Studies have reported that in the process of diabetic wound healing, the differentiation and proliferation abilities of epidermal cells are enhanced with the increase in Wnt and β-catenin expression, which accelerates wound healing [41].

#### 3.4.1 The Wnt pathways can affect wound healing by affecting extracellular matrix remodeling

Gay et al. [42] investigated the relevance between macrophages and fibrotic scars by establishing the wound-induced hair neogenesis (WIHN) in rat model, and the results showed that chronic Wnt activity in the WIHN model was correlated with fibrotic WIHN-scar formation, and fibrotic skin healing was achieved by the way that late macrophage devoured SFRP4, an inhibitor of Wnt, to drive chronic Wnt activity. Hu et al. [43] inquired into the effects of ganoderma lucidum polysaccharides (GL-PS) on wound healing by establishing a human skin fibroblast model and a mouse full-thickness dermoplasty model, and the results indicated that GL-PS up-regulated the expression of Wnt/β-catenin and TGF-β and boosted the viability and migration ability of fibroblasts, which promoted the wound healing rate and shortened the healing time.

#### 3.4.2 The Wnt signaling pathway can affect wound healing by affecting cell proliferation and repair

He et al. [44] studied the mechanism of MalAT1-containing exosomes derived from adipose-derived stem cells (ADSC-ExOS) amid wound healing through *in vitro* experiments. The results manifested that ADSC-ExOS containing MALAT1 could significantly promote cell proliferation and migration and inhibit cell apoptosis, and in turn induce wound healing by activating target Mir-124 in the Wnt/β-catenin pathway.

### 3.5 NF-κB signaling pathway

As an important pathway that causes inflammatory response in the body, the NF-κB signaling pathway is widely involved in various inflammatory processes and plays an important role in the occurrence and treatment of various diseases. After transferring to the nucleus, the activated NF-κB binds to the promoter or enhancer region of target gene, to induce the production of adhesion molecules in ECs and enhance the inflammatory response of leukocytes and the proliferation of fibroblasts, thus exerting pro-inflammatory effects [45,46,47].

#### 3.5.1 The NF-κB signaling pathway can affect wound healing by influencing inflammatory responses

Kong et al., through *in vivo* and *in vitro* experiments, discovered that the inhibition of NF-κB p65 nuclear translocation could suppress the inflammatory phenotype conversion of vascular smooth muscle cells (VSMCs), lighten inflammation responses, and promote angiogenesis. Sangiovanni et al. [48] explored the mechanism of *Cannabis sativa* L. ethanolic extract (CSE) in skin inflammation and wound injury through *in vitro* experiments. The results showed that CSE could inhibit TNF-α-induced NF-κB-driven transcription, and IL-8 and MMP-9 release, and exert anti-inflammatory activity, so as to promote wound healing. Chen et al. [49] studied the therapeutic effect and mechanism of thalidomide on rosacea-like mouse skin model induced by LL37. The results manifested that the overexpression of NF-κB in rosaceas led to distinct inflammatory reaction, and at the same time, thalidomide improved skin inflammation and promoted skin healing by inhibiting NF-κB expression. Romana-Souza et al. [50] studied the restorative effect of caffeic acid phenethyl ester (CAPE) on pressure ulcers through *in vivo* experiments, and the results showed that NF-κB was involved in the inflammatory reaction of pressure ulcers, leading to chronic inflammation and delaying wound closure, and CAPE might reduce the activation of NF-κB P65 and promote wound healing [51,52,53,54,55].

#### 3.5.2 The NF-κB pathways can affect wound healing by affecting vascular regeneration

Studies have shown that VEGF transcription is regulated by the activation of the NF-κB signaling pathway; therefore, inhibiting the activation of the NF-κB signaling pathway can significantly inhibit VEGF expression and capillary formation [56].

### 3.6 Notch signaling pathway

Notch pathway does not transfer signals by gradual activation of kinase phosphorylation, but it releases the Notch protein fragments (NICD or ICN) with transcriptional regulatory activity through three-step protease hydrolysis and then binds them to the transcription factor CSL to regulate downstream gene expression. Notch signaling pathway is closely related to wound healing [57,58,59,60].

#### 3.6.1 The Notch pathways can affect wound healing by affecting vascular regeneration

Li et al. [61] constructed a transgenic zebrafish model to study the impact of tetraspanin 18 (Tspan18) on angiogenesis, and the results demonstrated that arterial venous specification was mainly regulated by the Notch pathway, Tspan18 was expressed in blood vessels, and in addition, Notch and VEGF were regulated to promote angiogenesis and wound healing.

#### 3.6.2 The Notch signaling pathway can affect wound healing by influencing inflammatory responses

He et al. used the RBP-J gene down-regulated by the Notch signaling to establish a wound inflammation model. In the experimental process, it was found that the blockade of the Notch signaling pathway could reduce the expression of inflammatory cytokines such as TGF-β1, CCL2, and TNF-α in macrophages and the infiltration of inflammatory cells, thus inhibiting the formation of pathological scars and promoting wound healing [62,63,64].

#### 3.6.3 The Notch pathways can affect wound healing by affecting extracellular matrix remodeling

Ebrahim et al. verified that the joint regulation of platelet-rich plasma therapy and adipose-derived mesenchymal stem cell therapy effectively promoted wound re-epithelialization and granulation tissue formation in diabetic rats by regulating the Notch pathway, and the collagen area percentage, epidermal thickness, and angiogenesis were also significantly increased thereby [65,66].

### 3.7 Hippo signaling pathway

The Hippo pathway consists of a group of conserved kinases. After sensing the signals from the extracellular environment, the upstream membrane protein receptors undergo a series of kinase phosphorylation reactions and finally act on the downstream effectors YAP and TAZ. YAP/TAZ has the function of transcriptional regulation, which can fine-regulate the cell phenotype. Moreover, the Hippo pathway regulates cell proliferation, differentiation, and apoptosis by acting on the downstream effectors YAP and TAZ [67].

#### 3.7.1 The Hippo pathways can affect wound healing by affecting extracellular matrix remodeling

Mascharak et al. [68] probed into the connection between ENF and scar formation by establishing cell transplantation and transgenic mouse models, and the results indicated that mechanical tension drove Engrailed-1 (EN-1) activation through typical mechanical transduction signals, and inhibition of YAP might block En1 activation *in vivo* to reduce scar production and promote wound regeneration and healing through ENF. Brewer et al. [69] found that the scarless regenerative wound healing characteristic of spiny mice was related to the target protein YAP in the Hippo signaling pathway and activation of YAP *in vitro* could prevent fibrosis and provide a new idea for promoting scarless regenerative wound healing *in vivo*.

#### 3.7.2 The Hippo signaling pathway can affect wound healing by affecting cell proliferation and repair

Yuan et al. [70] demonstrated through *in vivo* and *in vitro* experiments that YAP1/TaZ-Tead inhibition would destroy skin homeostasis and lead to skin ulceration in mice. Shome et al. [71] found that the upregulation of Hippo transcription factor YAP and its downstream effectors CTGF and Cyr61 drove paracrine signals in the mechanism of cold atmospheric pressure plasma treatment for wound healing, which facilitated to cell migration and accelerated wound healing.

## 4 Crosstalk between signaling pathways

### 4.1 PI3K/AKT and MAPK synergy in angiogenesis

**Figure 2:**
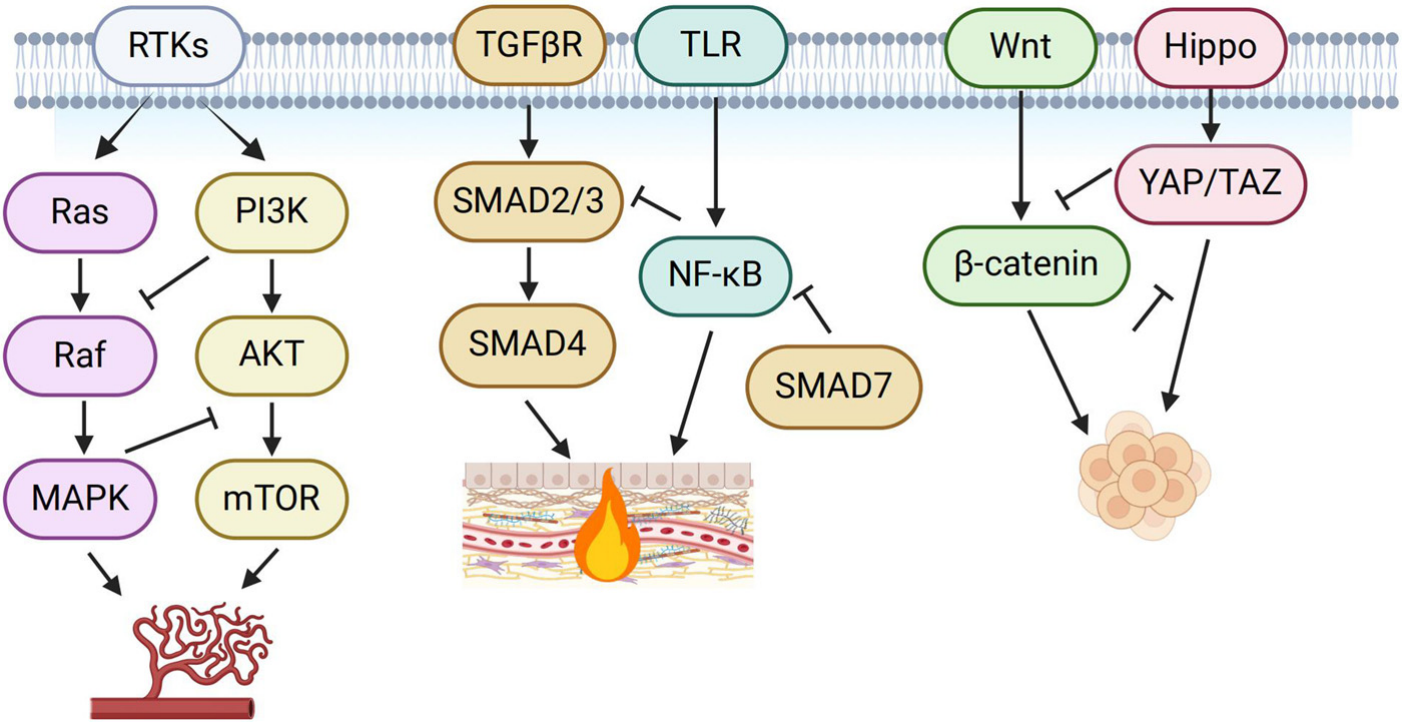
Schematic diagram showing cross-talk between signaling pathways implicated in chronic wound healing.

The interplay between the PI3K/AKT and MAPK signaling pathways is crucial for regulating angiogenesis in wound healing (Figure 2). PI3K/AKT signaling is primarily activated by receptor tyrosine kinases such as VEGFR2 and FGFR, leading to the phosphorylation of AKT, which stabilizes HIF-1α and enhances VEGF expression. Meanwhile, the MAPK/ERK pathway promotes EC proliferation and migration by activating downstream effectors such as matrix metalloproteinases and endothelial nitric oxide synthase.

Recent studies highlight that PI3K/AKT and MAPK pathways exhibit bidirectional regulation at multiple levels. PI3K/AKT can inhibit Raf phosphorylation, thereby downregulating MAPK signaling under certain conditions [72]. Conversely, ERK activation can phosphorylate and suppress TSC2, relieving mTOR inhibition and boosting AKT activity. This reciprocal regulation ensures a controlled balance between endothelial proliferation and vessel maturation [73].

In chronic wounds, prolonged hyperglycemia and oxidative stress impair PI3K/AKT activation, reducing VEGF-mediated angiogenesis. Simultaneously, sustained activation of MAPK, particularly the JNK and p38 branches, leads to cell apoptosis and excessive inflammatory signaling, further hindering capillary formation [74]. Moreover, the dysregulation of upstream regulators such as integrins and focal adhesion kinase disrupts cross-signaling between PI3K/AKT and MAPK, compounding angiogenic failure. Targeting these pathways simultaneously, for example, by using PI3K agonists alongside controlled MAPK inhibition, may help restore vascular homeostasis and improve wound healing [10].

### 4.2 NF-κB and TGF-β interplay in inflammation and extracellular matrix remodeling

The NF-κB and TGF-β signaling pathways exhibit intricate crosstalk in the regulation of inflammation and extracellular matrix remodeling. NF-κB is primarily activated by inflammatory stimuli via toll-like receptors, leading to increased transcription of cytokines such as TNF-α and IL-1β. Meanwhile, TGF-β is involved in the later stages of wound healing, facilitating fibroblast activation and extracellular matrix synthesis through Smad2 and Smad3-dependent transcriptional regulation.

Recent insights suggest that NF-κB and TGF-β pathways regulate each other through multiple feedback loops. NF-κB suppresses Smad3 activity, reducing TGF-β-mediated fibroblast differentiation and extracellular matrix production. In contrast, TGF-β can inhibit NF-κB signaling via Smad7, which interferes with IκB kinase activation, thereby limiting inflammatory responses [75]. However, in chronic wounds, persistent NF-κB activation prevents the transition to the proliferative phase, leading to prolonged inflammation and excessive matrix metalloproteinase expression, which degrades extracellular matrix components [76]. Additionally, TGF-β’s role in fibrosis is exacerbated in chronic wounds due to an imbalance in Smad-independent pathways, particularly the p38 MAPK and PI3K/AKT branches. Excessive TGF-β signaling leads to myofibroblast overactivation and excessive extracellular matrix deposition, contributing to fibrosis [77]. Furthermore, recent findings highlight that microRNAs, such as miR-146a and miR-200b, play a role in modulating the NF-κB-TGF-β interaction by regulating key signaling intermediates such as TRAF6 and Smad4 [72]. Therapeutic interventions targeting microRNAs or modulating the NF-κB–TGF-β balance may provide novel approaches to controlling inflammation and fibrosis in chronic wound healing.

### 4.3 Wnt/β-catenin and Hippo signaling crosstalk in cell proliferation

The interplay between Wnt/β-catenin and Hippo signaling is crucial for regulating keratinocyte and fibroblast proliferation during wound healing. Wnt/β-catenin signaling promotes cell proliferation by stabilizing β-catenin, allowing it to translocate into the nucleus and activate target genes involved in cell cycle progression. In contrast, Hippo signaling functions as a growth suppressor by phosphorylating and inactivating YAP and transcriptional coactivator with PDZ-binding motif (TAZ), thereby preventing excessive cell proliferation.

Crosstalk between these pathways occurs at multiple regulatory points. Hippo signaling components, particularly large tumor suppressor kinases (LATS1/2), can phosphorylate YAP/TAZ, promoting their cytoplasmic retention and degradation. This suppresses β-catenin activity, as YAP/TAZ are known to interact with β-catenin to enhance its nuclear translocation and transcriptional activation of Wnt target genes [78]. Conversely, Wnt signaling inhibits Hippo pathway activation by suppressing GSK-3β, which not only stabilizes β-catenin but also promotes YAP/TAZ activity. Additionally, YAP has been shown to bind to disheveled (DVL), a key mediator of Wnt signaling, further enhancing β-catenin-driven transcription [79]. In the context of wound healing, disruption of this crosstalk contributes to impaired tissue regeneration. Persistent inflammation and oxidative stress often suppress Wnt signaling, leading to reduced β-catenin activity and delayed keratinocyte migration. Simultaneously, aberrant Hippo pathway suppression can result in uncontrolled YAP/TAZ activation, driving excessive fibroblast proliferation and ECM deposition, ultimately leading to fibrosis and scar formation. This imbalance hinders proper wound closure and functional tissue restoration [72].

Targeting the Wnt/β-catenin-Hippo signaling crosstalk presents a promising therapeutic strategy. Enhancing Wnt signaling while fine-tuning Hippo pathway activity could promote controlled cell proliferation and re-epithelialization without exacerbating fibrosis. Small-molecule activators of Wnt or inhibitors of LATS1/2 could help restore β-catenin and YAP/TAZ activity in a regulated manner, fostering an optimal wound healing environment.

## 5 Therapeutic strategies targeting key signaling pathways in wound healing

Effective wound healing requires precise regulation of signaling pathways involved in inflammation resolution, angiogenesis, cell proliferation, and extracellular matrix remodeling. Dysregulation of these pathways in wound healing contributes to delayed healing and pathological tissue remodeling. Recent advances in targeted therapies, including small-molecule inhibitors, gene therapy, and biologics, have shown promise in modulating these pathways to restore normal wound healing dynamics. This section summarizes current therapeutic approaches targeting PI3K/AKT, MAPK, NF-κB, TGF-β, Wnt/β-catenin, and Hippo signaling.

### 5.1 Small-molecule inhibitors

Small-molecule inhibitors have been extensively investigated for their ability to modulate key signaling pathways in chronic wound healing. The PI3K/AKT pathway plays a central role in angiogenesis and cell survival, making it a potential target for wound therapy. Small-molecule agonists, such as SC79, an AKT activator, have been shown to promote EC proliferation and enhance vascular regeneration in preclinical wound models [80]. Conversely, excessive MAPK activation, particularly through the JNK and p38 branches, can contribute to inflammation-induced apoptosis. p38 inhibitors such as SB203580 have demonstrated efficacy in reducing inflammation and improving wound closure by preventing excessive fibroblast senescence [81].

Persistent NF-κB activation in chronic wounds sustains an inflammatory microenvironment, while excessive TGF-β signaling promotes fibrosis. Small-molecule NF-κB inhibitors have been explored for their ability to downregulate pro-inflammatory cytokines [82]. In contrast, selective TGF-β modulators, including losartan and pirfenidone, have been investigated for their antifibrotic properties, promoting balanced extracellular matrix remodeling [83,84].

Wnt activators such as lithium chloride and CHIR99021 enhance cellular proliferation and migration by stabilizing β-catenin [85,86]. Meanwhile, Hippo pathway inhibitors, such as verteporfin, reduce excessive fibroblast proliferation by preventing YAP/TAZ nuclear translocation, thus limiting fibrosis [87].

### 5.2 Gene therapy

Gene therapy has emerged as a promising strategy for directly modulating key signaling pathways involved in chronic wound pathophysiology. Viral and non-viral gene delivery strategies have been employed to enhance AKT activity in chronic wounds. Gene therapy using VEGF-A-expressing collagen-mimetic peptide tethers has been shown to activate the PI3K/AKT pathway, promoting angiogenesis and accelerating wound closure [88]. In parallel, CRISPR/Cas9-mediated editing of TGF-β receptors has been investigated for selectively modulating fibrotic responses while preserving its role in extracellular matrix deposition [89]. Overexpression of Wnt ligands or β-catenin-stabilizing constructs has been shown to improve epithelial regeneration in chronic wounds. Conversely, knockdown of YAP using shRNA-based gene therapy has been explored to prevent fibroblast hyperproliferation and excessive scarring [90].

### 5.3 Biologic therapy

Recombinant VEGF and PDGF have been used to enhance angiogenesis by stimulating PI3K/AKT and MAPK pathways, though their efficacy has been variable due to rapid degradation in the wound microenvironment. Encapsulation strategies using nanoparticles have been developed to enhance the stability and delivery efficiency of these growth factors [91].

Monoclonal antibodies targeting pro-inflammatory cytokines, such as anti-TNF agents (infliximab) and IL-6 inhibitors (tocilizumab), have been investigated for modulating NF-κB-mediated inflammation. Meanwhile, TGF-β-neutralizing antibodies, such as fresolimumab, have been tested for preventing excessive fibrosis in chronic wounds [92].

Exosomes derived from stem cells have gained attention for their ability to modulate multiple signaling pathways simultaneously. Mesenchymal stem cell-derived exosomes have been shown to enhance diabetic wound healing and skin regeneration [93].

## 6 Conclusion and future directions

Wound healing arises from the complex dysregulation of multiple signaling pathways, including PI3K/AKT, MAPK, NF-κB, TGF-β, Wnt/β-catenin, and Hippo. While significant progress has been made in understanding their roles in wound healing, several challenges remain. The intricate crosstalk between these pathways is not yet fully elucidated, making it difficult to develop precise therapeutic strategies that effectively restore wound healing without unintended side effects. Existing treatments, such as growth factors and anti-inflammatory agents, often suffer from limited efficacy due to rapid degradation, poor bioavailability, and non-specific targeting. Although preclinical studies on small-molecule inhibitors, gene therapy, and biologics have demonstrated promising results, the translation of these findings into clinical practice remains challenging due to safety concerns, inconsistent outcomes, and a lack of large-scale clinical validation. Moreover, patient variability, including differences in wound etiology, metabolic conditions, and genetic predispositions, complicates the development of universally effective therapies.

Future research should focus on elucidating the dynamic interactions between these pathways using advanced multi-omics technologies, such as single-cell RNA sequencing and proteomics, to better understand how signaling networks contribute to impaired healing. Instead of targeting single pathways, combination therapies that fine-tune multiple signaling cascades may offer better outcomes by addressing both inflammation and tissue regeneration while preventing fibrosis. Advances in nanotechnology-based drug delivery systems, including hydrogels and biomaterials, hold great potential for improving the stability, bioavailability, and targeted release of therapeutic agents at the wound site. Additionally, personalized medicine approaches, guided by artificial intelligence and machine learning, could help stratify patients based on molecular profiles, enabling precision-targeted therapies tailored to individual wound characteristics. Finally, bridging the gap between preclinical research and clinical application will require well-designed clinical trials that assess the long-term efficacy and safety of emerging therapies, optimize dosing strategies, and minimize adverse effects. By addressing these challenges, future research can pave the way for more effective and personalized treatments for wound healing, ultimately improving patient outcomes and reducing healthcare burdens.
